# Factor Structure and Measurement Invariance of the Spanish Burnout Inventory Among Professionals Across 17 Countries and Regions

**DOI:** 10.1007/s11482-022-10108-1

**Published:** 2022-11-03

**Authors:** Pedro R. Gil-Monte, Begoña Espejo, Irene Checa, Pedro Gil-LaOrden, Kezia Angeline J, Mary Sandra Carlotto, Daniela Converso, Ángel Deroncele-Acosta, Hugo Figueiredo-Ferraz, Laura Galarza, Viviola Gómez-Ortiz, Ester Grau-Alberola, Javier Labarthe, Marta Llorca-Pellicer, Christy Mekala V, Alejandra Misiolek-Marín, Eldis Román-Cao, Edwin Salas-Blas, Sandrine Schoenenberger, Sara Unda-Rojas, Sara Viotti

**Affiliations:** 1 Unidad de Investigación Psicosocial de la Conducta Organizacional (UNIPSICO), Valencia, Spain; 2 Faculty of Psychology (Dep. Methodology of Behavioral Sciences), University of Valencia, Av. Blasco Ibáñez, 21; 46010, Valencia, Spain; 3 St. John’s Research Institute, Bangalore, South India India; 4 Universidade do Vale do Rio dos Sinos, Cristo Rei, Brazil; 5 Università degli Studi di Torino, Turin, Italy; 6 Universidad César Vallejo, Lima, Peru; 7 Universidad Internacional de Valencia (VIU), Valencia, Spain; 8 Universidad de Puerto Rico, San Juan, Puerto Rico; 9 Universidad de Los Andes, Bogotá, Colombia; 10 Universidad Internacional de La Rioja, Logroño, Spain; 11 Universidad Católica del Uruguay, Montevideo, Uruguay; 12 Bishops College of Nursing, Dharapuram, Tamil Nadu India; 13 Clínica Art Psychology and Psychotherapy, Barcelona, Spain; 14 Universidad Técnica de Manabí, Portoviejo, Ecuador; 15 Universidad Peruana de Ciencias Aplicadas, Lima, Peru; 16 Université Catholique de Lille, Lille, France; 17 Université de Lorraine), Metz, France; 18 Universidad Nacional Autónoma de México, FES Zaragoza, Ciudad de Mexico, Mexico

**Keywords:** Spanish Burnout Inventory, confirmatory factor analysis, construct validity, measurement invariance, alignment method, cross-cultural validity

## Abstract

Studies on the prevalence of burnout in professionals in service organizations who work in direct contact with the clients or users of the organization have concluded that burnout is a serious health disorder that has increased due to the COVID-19 pandemic. A significant advantage of the Spanish Burnout Inventory (SBI) over other instruments is that it provides a broader conceptualization of burnout by including feelings of guilt as a dimension of burnout to explain its development. However, the measurement invariance of the SBI across countries has not been investigated. The purpose of this study was to test the measurement invariance of the SBI among professionals across 17 countries and regions in Europe, Latin America, and Asia, and in different languages. All the countries showed a good fit to the four-factor model, except the Indian sample, which was excluded from the measurement invariance study. Using the alignment method, it was possible to verify the scalar measurement invariance of the four SBI factors across 15 countries and one Spanish region (16 samples). The comparison of estimated latent means indicates that France is the country with the lowest scores on the Enthusiasm factor and the highest scores on the negative factors (Exhaustion, Indolence, and Guilt). In contrast, the Andean countries, Colombia, Peru, and Ecuador, show the highest latent means on the Enthusiasm factor and the lowest means on the negative factors. These results support the validity of the SBI in the countries and regions in Europe and Latin America included in this study.

The ICD-11 (World Health Organization, 2019) defines burnout syndrome as a psychological response to chronic work-related stress that has not been successfully managed. It is a nonpsychiatric health disorder characterized by three dimensions: (1) feelings of exhaustion; (2) increased mental distance from one’s job; and (3) a sense of ineffectiveness. Burnout refers specifically to phenomena in the occupational context and should not be used to describe experiences in other areas of life. It has been classified (QD85) as a problem associated with employment or unemployment.

Burnout can be considered a significant worldwide occupational health problem that appears mainly in professionals in service organizations who work in direct contact with the clients of the organization (Gil-Monte, 2005), and it can be expressed as psychological symptoms related to depressive mood (Figueiredo-Ferraz et al., 2021; Gil-Monte, 2012; Parker & Tavella, 2021). Interest in the study of burnout and the health problems associated with its development has grown in the past decade due to social and occupational changes that have led to an increase in work stress along with social demands to increase the quality of working life and the prevention of psychosocial risks at work (Eurofound, 2016; 2018). The 2021 Work and Well-being Survey by the APA found that 40% of adults who perform job activities related to customer interaction, entertainment, sales, or other services-oriented work felt high levels of emotional exhaustion (American Psychological Association, 2022). In the 2022 trends report, the APA includes burnout as one of the emerging trends, reporting that burnout and stress are everywhere. Both are at all-time highs across professions, and among health care workers they are exacerbated by the unrelenting stressors associated with the COVID-19 pandemic (Abramson, 2022).

According to the literature review carried out by Eurofound (2018), there are variations in burnout levels, and severe forms of burnout are infrequent, with less than 10% reporting symptoms of depression, incapacity to work, or psychosomatic disorders. More moderate forms of burnout were reported by between 15% and 25% of respondents in the different studies. Systematic literature reviews and meta-analyses carried out in healthcare professionals have concluded that burnout prevalence: (a) among physicians practicing in Europe has been estimated at 7.7% (Hiver et al., 2022); (b) among nurses worldwide was 10%, with the highest prevalence in Intensive and Critical Care nurses (14.4%) (Woo et al., 2020); (c) ranges from 3 to 66% in healthcare professionals providing palliative care, with most studies reporting a burnout prevalence of 18% or more (Dijxhoorn et al., 2021); and (d) was 47.3% in postgraduate medical trainees (Naji et al., 2021). In Menscape’s 2021 National Physician Burnout & Suicide Survey, 42% of the physicians reported burnout, out of a sample of more than 12,000 American physicians, with the highest prevalence found in Critical Care physicians (51%) (Medscape, 2021).

Studies of burnout prevalence in educators have concluded that burnout in teachers is a serious problem that has increased during the COVID-19 pandemic (Fernández-Suárez et al., 2021; Weißenfels et al., 2022) due to concern about unsafe school conditions and the pressure of virtual work (Pressley et al., 2021). Working conditions in education (Gil-Monte et al., 2011; Kim et al., 2021) have turned teaching into a profession with a high risk of developing this syndrome. Teaching requires high levels of commitment to the job, and most schools do not consider the work-life balance of their teachers and have unrealistic expectations of them. Instead, school leaders relentlessly focus on improvement and external inspections, accountability, and a blame culture, which discourages teaching staff from revealing how they feel and increases stress at work. Results from systematic reviews showed that the rate of overall burnout was close to 24% in physical education teachers (Alsalhe et al., 2021) and 37% in university teachers (Fernández-Suárez et al., 2021). In addition, Carlotto & Câmara (2019) estimated the prevalence of critical burnout in Brazilian teachers from public elementary schools at 25.8%, and Li et al., (2020) estimated it at 53.20% in Chinese preschool teachers. However, the prevalence was lower in Finnish primary and secondary school teachers, ranging from 3.7 to 5.6% (Pyhältö et al., 2021).

The symptoms of burnout syndrome according the definition of the World Health Organization (2019) can be assessed using the Maslach Burnout Inventory (MBI; Maslach & Jackson 1981; Maslach et al., 1996), because those symptoms are the three dimensions or subscales of this questionnaire -i.e., Emotional exhaustion or Exhaustion, Depersonalization or Cynicism and Personal accomplishment or Professional Efficacy (Maslach & Leiter, 2017; Maslach et al., 1996), but a review of the literature indicates that researchers have been troubled by some of the limitations of the MBI (Kristensen et al., 2005), for example: (a) researchers have proposed different and not always matching solutions for the factor structure (e.g., two-factor model instead of the original three-factor model) (Loera et al., 2014), (b) the MBI dimensions were not theoretically deduced before construction of the questionnaire; instead, they were labeled after the factor analysis (Schaufeli & Van Dierendonck, 1993), (c) it is necessary to more broadly capture the nature of burnout (Gil-Monte et al., 2013) because the definition of burnout according the MBI is based on a limited concept of burnout (Halbesleben & Demerouti, 2005), (d) the MBI was developed as a research tool, not as a diagnostic one (Doulougeri et al., 2016).

Most of the studies on the prevalence of burnout have been carried out with psychometric instruments that do not differentiate between burnout profiles. However, a literature review allows us to conclude that burnout may progress in different ways, and there is empirical evidence for differentiating between types or profiles of burnout (Gillet et al., 2020; Guidetti et al., 2018b; Leiter & Maslach, 2016; Pyhältö et al., 2021; Salmela-Aro & Upadyaya, 2020) related to predictors (Llorca-Pellicer et al., 2021) and consequences (Misiolek-Marín et al., 2020). These profiles could be explained by the fact that some professionals develop higher clinical patterns of burnout, personal distress, and diminished performance as an end state of burnout, whereas others remain in the organization for years without developing relevant personal problems due to work-related stress, but they have attitudes and behaviors of indolence and distance from their job (Figueiredo-Ferraz et al., 2021; Gil-Monte, 2012; Rabasa et al., 2016). Therefore, studies in the field of burnout should evaluate burnout by taking into consideration different types of burnout and their consequences.

## Overview of the Spanish Burnout Inventory

The Spanish Burnout Inventory (SBI) (Gil-Monte, 2019) is one of the most extensively applied questionnaires to evaluate burnout in Latin America (Díaz & Gómez, 2016) and it has been applied in different countries of Europe to assess job burnout: Czech Republic (Alföldy & Gil-Monte, 2010), Germany (Bosle & Gil-Monte, 2010), Italy (Guidetti et al., 2018b), Poland (Misiolek et al., 2017), Portugal (Figueiredo-Ferraz et al., 2013) and United Kingdom (Cramer et al., 2020). It was developed to address the problems associated with the MBI and other questionnaires assessing burnout.

The theoretical model of burnout developed by Gil-Monte (2005) is based on the concept that burnout is a response to chronic job stress that stems primarily from problematic interpersonal work relationships characterized by four symptoms: (1) cognitive deterioration (i.e., low enthusiasm toward the job), (2) emotional deterioration (i.e., psychological exhaustion), (3) attitudes and behaviors of indifference, indolence, withdrawal, and in some cases, (4) feelings of guilt. The SBI comprises 20 items divided into four subscales: (1) Enthusiasm toward the job: the individual’s desire to achieve goals at work because it is a source of personal pleasure. This scale is similar to that of the Personal accomplishment of the MBI. (2) Psychological exhaustion: the appearance of emotional and physical exhaustion due to the fact that he or she must deal daily with people at work who present problems. This scale is similar to that of the Emotional exhaustion of the MBI. (3) Indolence: the appearance of negative attitudes of indifference and cynicism toward the organization’s clients. This scale is similar to that of the Depersonalization of the MBI. (4) Guilt: the appearance of feelings of guilt about negative attitudes developed on the job, especially toward the people with whom he or she establishes work relationships. It is a new dimension added to the concept of burnout (Gil-Monte et al., 2013; Maslach & Leiter, 2017) to explain different types of burnout, considering the role of guilt feelings in the relationship between burnout and its consequences.

The model of the SBI identifies two profiles in the development of burnout. In both, attitudes and behaviors of indolence are understood as a coping strategy that arises to deal with emotional and cognitive deterioration. For some professionals, this coping strategy allows them to manage the levels of strain, but other professionals find this way of proceeding to be inadequate and develop feelings of guilt. Profile 1 is characterized by low enthusiasm toward the job, high levels of psychological exhaustion, and indolence. Individuals who fit Profile 1 suffer moderately from work-related stress, and they are able to do their work without experiencing very high feelings of guilt (i.e., critical levels). However, Profile 2 is characterized by more severe manifestations of burnout and the use of indolence as a dysfunctional coping strategy. Individuals who fall into Profile 2 feel they cannot do their jobs properly, which makes them develop greater feelings of guilt and then other health disorders, such as symptoms of depression, and absenteeism (Figueiredo-Ferraz et al., 2021; Gil-Monte, 2012).

The SBI fits the common language (in terms of measurement) for the study of burnout delimited by the MBI, which is the most widely used instrument to measure burnout in different cultures and languages (Díaz & Gómez, 2016; Lheureux et al., 2017; McCormack et al., 2018; Rotenstein et al., 2018). However, it adds the dimension of Guilt to improve the diagnosis of job burnout by identifying the profiles.

Taking into consideration the SBI factor structure, previous exploratory factorial analysis studies have shown a four-factor structure similar to that of the model, representing Enthusiasm toward the job, Psychological Exhaustion, Indolence, and Guilt (Bosle & Gil-Monte, 2010; Esteves et al., 2020; Olivares & Gil-Monte, 2007). Results have been replicated through confirmatory factor analysis, obtaining empirical support for the four-factor structure model across countries and occupational groups in several Latin American countries: Brazil (Carlotto et al., 2015), Chile (Olivares-Faúndez et al., 2018), Colombia (García et al., 2022), and Mexico (Gil-Monte et al., 2013); and in some European countries: Germany (Bosle & Gil-Monte, 2010), Italy (Viotti et al., 2015), Poland (Misiolek et al., 2017), Portugal (Figueiredo-Ferraz et al., 2013), and Spain (Gil-Monte & Manzano-García, 2015). In addition, systematic reviews and meta-analysis studies have concluded that the SBI possesses adequate psychometric properties for the study of burnout (Serna et al., 2018). However, to date, the measurement invariance of the SBI across countries has not been tested. This study aims to close this research gap because studies of invariance across countries provide greater support for the construct validity in different cultural groups, and they contribute to enhancing the validity and applicability of the SBI internationally.

## The Present Study

The purpose of this study was to test whether the SBI shows measurement invariance among professionals across 17 countries and regions. It aims to fill the gap in the literature by examining the measurement invariance of the SBI across different cultural contexts and languages in Latin America, Europe, and Asia. When a measure is invariant, it can be concluded that the same constructs are evaluated and construed in the same way in the different samples and, therefore, can be compared (Putnick & Bornstein, 2016).

## Method

### Participants and Procedures

The sample included 18,611 participants (*M* = 42.3 years, *SD* = 10.4, Age Range = 18–82 years; 73.4% female) from 17 countries and regions. Two samples from Spain are presented because one of them (Valencia) is a specific region of Spain were, in addition to Spanish, another language (Catalan) is spoken. Data from the questionnaire adapted to Catalan were obtained in this region (Llorca-Rubio et al., 2021). Regarding the working sector, 80.6% of the sample (*n* = 15,000) came from education, 12.5% (*n* = 2,334) from health, 1.5% (*n* = 277) from the disability sector, and 5.4% (*n* = 1,000) from other sectors. Regarding the type of contract, 75% of the sample (*n* = 11,273) were tenured staff, 23.7% (*n* = 3,566) were temporary staff, and 1.2% (*n* = 185) had another type of contract. The mean years of seniority in the profession of all the participants was 13.2 years (*SD* = 10.4), with a range in seniority of between 0.02 and 54 years of seniority. Sociodemographic data of the different countries and regions is shown in Table 1

**Table 1:** *Sociodemographic Characteristics of the 17 Countries and Regions*

Country		Language	Age			Gender	Working sector	Type of contract			Seniority in the profession (years)		
	*n*		*M*	*SD*	Range	% Female		% Tenured	% Temporary	% Other	*M*	*SD*	Range
Argentina	302	Spanish	40.6	9.1	21–66	87.1	Education	64.5	35.5	-	13.9	9.2	0.08-38
Brazil	1,943	Portuguese	42.4	10.4	19–73	75.5	Education	68.9	31.1	-	13.8	9.3	0.50–48
Chile	741	Spanish	35.5	8.3	19–69	71.9	Disability (37.4%) / Other (62.6%)	95.9	4.1	-	9.8	8.1	0.02-44
Colombia	675	Spanish	38.7	9.4	18–82	63.3	Health	69.0	31.0	-	13.1	8.3	1.00–36
Costa Rica	477	Spanish	-	-	-	38.8	Other	-	-	-			-
France	316	French	45.7	9.7	25–64	83.2	Education	90.2	9.8	-	18.6	9.7	0.06-41
Italy	891	Italian	45.9	9.4	22–64	89.1	Education	79.5	20.5	-	20.6	11.3	0.02-43
Spain	2,750	Spanish	45.6	9.2	23–67	74.2	Education	99.5	0.5	-	18.1	9.8	0.02-47
Poland	534	Polish	41.7	9.2	24–65	69.9	Health	52.9	25.3	21.8	16.0	9.1	1.00–40
Puerto Rico	736	Spanish	37.2	10.4	22–70	82.9	Education (27.6% / Health (72.4%)	-	-	-	11.4	9.7	1.00–44
Valencia (Spain)	1,228	Catalan	46.2	9.1	20–67	66.2	Education	99.5	0.5	-	17.5	10.2	0.02-45
Mexico	1,358	Spanish	42.2	9.9	21–77	72.2	Education	71.3	28.7	-	16.0	9.8	0.02-54
Peru	327	Spanish	47.0	10.6	22–75	72.2	Education	75.8	24.2	-	20.1	10.3	1.00–42
Portugal	684	Portuguese	36.7	9.7	22–68	74.5	Education (30.9%) / Health (69.1%)	81.1	16.1	2.8	13.5	9.5	0.02-37
Uruguay	4,734	Spanish	41.1	10.4	20–75	75.1	Education	54.0	44.2	1.8	6.4	7.4	0.02-42
India	210	Tamil (43.8%) / English (56.2%)	32.2	10.0	20–64	79.8	Education (15.3%) /Health (55.6%) / Other (29.1%)	-	-	-	8.7	9.2	0.25-42
Ecuador	705	Spanish	45.8	10.1	22–75	63.3	Education	79.0	21.0	-	16.0	10.0	1.00–51

*Note*. A hyphen is offered when the data was not retrieved.

The data collection procedure was as follows. First, the first author contacted a number of collaborators to invite them to participate in the study. In addition to Spain, fifteen countries were recruited through this process. Second, the first author chose some data that were used in previous published studies to contribute to the international validation of the SBI (Brazil, Chile, Colombia, Italy, Mexico, Poland, Portugal, and Spain). Finally, to further extend the number of countries included in the study, the collaborators send some data that had been collected for unpublished previous studies carried out in collaboration with the first author (Argentina, Costa Rica, India, Puerto Rico, and Uruguay) or specifically for this study (Ecuador, France, and Peru). The first author sent the SBI translation to the collaborators living in countries where the first language was not Spanish, and the questionnaire had not been validated (France and India). In these countries, prior to starting the data collection, the items on the SBI were translated and back translated. The two versions obtained were compared, discussed, and reviewed until complete agreement was reached among the collaborators and the first author.

Some data were collected by paper and pencil at the workplace in different cities in: Argentina, Brazil (Gil-Monte et al., 2010), Colombia (by aggregating different samples of healthcare professionals, e.g., Tejada & Gómez 2012), Costa Rica, Italy (e.g., Guidetti et al., 2018a; Guidetti et al., 2018b), Mexico, Poland (Misiołek et al., 2017), Portugal (Figueiredo-Ferraz et al., 2013), and Spain (both Spanish and Catalan languages) (e.g., Llorca-Pellicer et al., 2021). Other data were collected online around the country through Google Forms: Brazil (Diehl & Carlotto, 2020), Ecuador, France (the study was approved by the Research Ethics Committee of the Universidad Internacional de La Rioja (UNIR), Spain), and Puerto Rico. Data from Chile were collected by paper and pencil during non-working time at the workplace in two organizations in Valparaíso and Santiago (Gil-Monte & Olivares, 2011) and online by a specific application developed for previous studies. Data from India were collected by paper and pencil at the workplace (the researcher was present during the data collection) and online (Google Forms) in and around Tamil Nadu (Southern India). Data from Peru were collected online through Google Forms and Facebook (around 33% of the data) from teachers working in Lima and Cuzco. Some participants were contacted by Whatsapp, and then the researcher used traditional snowball sampling to increase the sample size. Data from Uruguay were collected online by a specific application similar to Survey Monkey developed for the “Instituto Nacional de Evaluación Educativa” (INEEd) (Ministry of Education and Culture, Government of Uruguay) to analyze the quality of working life in teachers across the country (https://www.ineed.edu.uy/nuestro-trabajo/bases-de-datos/488-encuesta-de-salud-ocupacional-docente-2019.html).

Participants received no reward for responding in any country or region. In all countries, the fundamental principles of the Declaration of Helsinki were respected (World Medical Association, 2013), with particular emphasis on the anonymity and confidentiality of the data collected and non-discrimination of participants. All the data collection procedures had a cross-sectional design, and participants were selected in a non-random manner.

### Instrument

The Spanish Burnout Inventory (Gil-Monte, 2019) comprises 20 items divided into four subscales: (1) Enthusiasm toward the job (5 items, e.g., *I see my job as a source of personal accomplishment*) (for the total sample in this study, α = 0.87, 95% CI [0.870, 0.876]); (2) Psychological exhaustion (4 items, e.g., *I feel emotionally exhausted*) (for the total sample, α = 0.86, 95% CI [0.856, 0.862]); (3) Indolence (6 items, e.g., *I think many students are unbearable*) (for the total sample, α = 0.77, 95% CI [0.763, 0.773]); and (4) Guilt (5 items, e.g., *I regret some of my behaviors at work*) (for the total sample, α = 0.81, 95% CI [0.805, 0.814]). Items are answered on a 5-point frequency scale, ranging from 0 (Never) to 4 (Very frequently: Every day). Low scores on Enthusiasm toward the job, together with high scores on Psychological Exhaustion and Indolence, as well as on Guilt, indicate high levels of burnout.

### Data Analysis

The psychometric and cross-cultural invariance study of the SBI was carried out following the guidelines established for this type of study (Asparouhov & Muthén, 2014; Byrne & van de Vijver, 2017). First, a confirmatory factor analysis (CFA) of the SBI was performed in each of the 17 samples studied. Specifically, the four-factor model, the model with the most support in the literature, was tested. Subsequently, in all the groups with an acceptable model fit, a multigroup confirmatory factor analysis (MGCFA) was carried out to test for configural, metric, and scalar invariance by countries or regions. The Robust Maximum Likelihood (MLR) estimator was used due to: (a) relatively small sample sizes in some regions, (b) the five-category response scale (Rhemtulla et al., 2012), and (c) potential deviations from normality (Yuan & Bentler, 2000). To examine model fit, the most common fit indices were used: Chi-Squared, Comparative fit index (CFI), Root-Mean-Square Error of Approximation (RMSEA), and Standardized-Root-Mean Square Residual (SRMR). The following cut-off points indicated an acceptable fit: CFI > 0.90, RMSEA < 0.06, SRMR < 0.08 (Brown, 2015; Hu & Bentler, 1999). Measurement invariance was examined by comparing the fit indices of the configural model and those of the scalar model. When sample size is adequate (total *N* > 300), a change of ≥ 0.010 in CFI, supplemented by a change of ≥ 0.015 in RMSEA or a change of ≥ 0.030 in SRMR, would indicate non-invariance (Chen, 2007).

Given that in most cross-cultural studies with a significant number of groups, scalar invariance is a big challenge (Cieciuch et al., 2019), the MGCFA with the alignment procedure was used to identify the most non-invariant parameters. This method recently received attention from cross-cultural research (Sawicki et al., 2022) because the alignment method makes it possible to optimize the reliable estimation of the means, despite the presence of a certain degree of measurement non-invariance (Byrne & van de Vijver, 2017). First, the free alignment approach procedure is tested. If scalar invariance is obtained by using this method, the estimation process ends. But if there is no scalar invariance, the fixed alignment approach is used. According to this method, proposed by Asparouhov & Muthén (2014), the country or region that shows the mean value of the factor closest to 0 will be specified as the reference group to test for measurement invariance. In the alignment optimization, three criteria are used to establish the existence of invariance. First, at most, 25% of the parameters must be non-invariant in order to consider the estimates of the means trustworthy (Cieciuch et al., 2019). Second, the contribution of each item (factor load or intercept) to the fit function is analyzed, and the lowest absolute value is considered the most invariant. Finally, the value of *R*^*2*^ is considered, which indicates the variation in these parameters between groups in the configural model that can be explained by the variation in the means of the factors and the variances between groups. According to Asparouhov & Muthén (2014), a value of *R*^*2*^ close to 1 indicates a high degree of invariance, whereas a value close to 0 suggests a low degree of invariance. Sociodemographic data have been obtained with SPSS. The other analyses have been carried out with Mplus 8.8(Muth?n & Muth?n, 2017).

## Results

The CFA of the four-factor model carried out with the complete sample showed a good fit to the data (χ^2^ (164) = 7,783.54, *p* < .001, CFI = 0.926, RMSEA = 0.050, RMSEA 90% CI [0.049, − 0.051], SRMR = 0.044). However, the CFA performed with the sample from each country or region (see Table 2) offered good results for all of them, except Argentina (label 1) and India (label 16), which showed fit indices below the cut-off point recommended as acceptable.

**Table 2:** Confirmatory Factor Analysis Results for the Four-Factor Model in Each of the 17 Countries and Regions in the Sample

Label	Country	χ^2^(164)	CFI	RMSEA	90% RMSEA CI	SRMR	Standardized factor loadings range
1	Argentina	348.48*	0.887	0.061	[0.052, − 0.070]	0.060	0.48-0.77
2	Brazil	967.57*	0.941	0.050	[0.047, − 0.053]	0.046	0.55-0.86
3	Chile	614.20*	0.918	0.061	[0.056, − 0.066]	0.064	0.46-0.88
4	Colombia	488.97*	0.913	0.054	[0.049, − 0.060]	0.043	0.47-0.81
5	Costa Rica	370.93*	0.919	0.051	[0.044, − 0.058]	0.052	0.33-0.83
6	France	329.05*	0.939	0.056	[0.048, − 0.065]	0.057	0.46-0.88
7	Italy	586.81*	0.926	0.053	[0.048, − 0.057]	0.048	0.44-0.83
8	Spain	1,136.04*	0.934	0.046	[0.044, − 0.049]	0.043	0.43-0.80
9	Poland	407.89*	0.928	0.053	[0.046, − 0.059]	0.052	0.50-0.82
10	Puerto Rico	577.34*	0.927	0.059	[0.053, − 0.064]	0.064	0.53-0.88
11	Valencia (Spain)	635.26*	0.923	0.048	[0.044, − 0.052]	0.044	0.43-0.82
12	Mexico	805.36*	0.921	0.054	[0.050, − 0.057]	0.045	0.46-0.85
13	Peru	326.22*	0.918	0.055	[0.046, − 0.064]	0.055	0.47-0.88
14	Portugal	460.34*	0.935	0.046	[0.041, − 0.052]	0.043	0.54-0.82
15	Uruguay	1,989.69*	0.940	0.048	[0.047, − 0.050]	0.047	0.50-0.85
16	India	415.85*	0.815	0.086	[0.075, − 0.096]	0.084	0.44-0.85
17	Ecuador	466.99*	0.926	0.051	[0.046, − 0.057]	0.055	0.45-0.84

*Note*. CFI = comparative fit index; RMSEA = root-mean-square error of approximation; CI = confidence interval; SRMR = standardized root-mean-squared residual

* *p* < .001

In the case of the Argentine sample, it can be seen that the RMSEA and SRMR values are adequate, whereas the CFI value does not exceed the cut-off point. However, the RMSEA and CFI values ​​can be inconsistent at times. Although these indices are commonly used to assess model fit, they do not produce comparable qualitative assessments across data sets because they are computed differently. RMSEA is a non-standardized fit index, and so arbitrary cut-off points are used for its interpretation. However, the CFI measures relative improvement in fit (Shi et al., 2019). Some authors point out that, although these two indices sometimes offer contradictory evaluations of the model, it does not mean that the model is poorly specified or that there is a problem with the data because they evaluate the fit of the model from different perspectives (Lai & Green, 2016). Likewise, in simulation studies, it has been observed that the RMSEA almost always rejects the model if the samples are large and the items’ response scale has five response categories. In contrast, the SRMR provides more reliable estimates of model fit. Because SRMR is a standardized fit index (compared to RMSEA, which is a non-standardized index), it shows greater power to reject models that show a poor fit to the data when the response scale is ordinal (as in this case), regardless of the number of parameters to be analyzed and the sample size (Shi et al., 2020). Based on these arguments, the fit of the estimated model in the Argentine sample could be evaluated by taking the SRMR values into consideration, which show a good fit to the data. Therefore, only the Indian sample was excluded from the measurement invariance study. Next, the estimation of the MGCFA was carried out with the 16 samples that offered good fit indices for the four-factor model. The results showed the existence of metric invariance (see Table 3), but they did not confirm the scalar invariance (CFI = 0.845, RMSEA = 0.070, SRMR = 0.076, ΔCFI = − 0.079, ΔRMSEA = 0.019, ΔSRMR = 0.021).

**Table 3:** Results of the Measurement Invariance Models

	χ²	*df*	Δχ²	Δ*df*	CFI	RMSEA	SRMR	ΔCFI	ΔRMSEA	ΔSRMR
Configural	10,416.29*	2624			0.932	0.051	0.048			
Metric	11,517.22*	2864	1,093.32*	240	0.924	0.051	0.055	− 0.008	0.000	0.007
Scalar	20,817.26*	3104	10,187.18*	240	0.845	0.070	0.076	− 0.079	0.019	0.021

*Note*. Δχ² = Chi-Square change; Δ*df* = degrees of freedom change; CFI = comparative fit index; RMSEA = root-mean-square error of approximation; SRMR = standardized root-mean-squared residual; ΔCFI = CFI change; ΔRMSEA = RMSEA change; ΔSRMR = SRMR change

* *p* < .001

Given that no scalar invariance was found, the alignment procedure was performed. After obtaining an unsatisfactory result with the free alignment approach, the fixed approach was tested. When observing the latent means of the 16 groups, it was concluded that the country with a combination closest to 0 in the four factors was Ecuador (Table 4, label 17). Therefore, the configural model was specified as a fixed alignment analysis with four factor means for Ecuador restricted to 0 and the factor means for the remaining 14 freely estimated countries. Table 4 shows the results of the alignment measurement for the 16 countries and regions finally considered for the analysis, the fit function contribution of both the factor loading and intercept for each item on the SBI, and *R*^*2*^.

**Table 4:** Results of the Alignment Measurement Invariance (non-invariance) of the Spanish Burnout Inventory over 16 Countries and Regions, Fit Function Contribution of both the Factor Loading and Intercept for each Item, and R^*2*^

	Factor loadings				Intercepts				All%
	Countries and regions	%	Fit function contribution	*R* ^*2*^	Countries and regions	%	Fit function contribution	*R* ^*2*^	
Factor Enthusiasm toward the Job		8.8				30.0			19.4
Item 1	1 2 **(3)** 4 5 6 7 **(8)** 9 10 11 12 13 14 **(15)** 17		-46.55	0.71	1 **(2)** 3 **(4) (5) (6) (7) (8)** 9 10 **(11)** 12 13 **(14) (15)** 17		-51.37	0.84	
Item 5	1 2 **(3)** 4 5 6 7 8 9 10 11 12 13 14 15 17		-42.35	0.90	1 2 3 4 5 6 **(7) (8)** 9 10 **(11)** 12 13 14 **(15)** 17		-45.93	0.95	
Item 10	1 2 **(3)** 4 5 6 7 8 9 **(10)** 11 **(12)** 13 14 15 17		-43.59	0.84	1 2 3 4 5 6 7 8 9 10 11 12 13 14 15 17		-41.97	0.95	
Item 15	1 2 3 4 5 6 7 8 9 10 11 12 13 14 15 17		-42.11	0.77	1 2 **(3)** 4 5 **(6)** 7 8 9 **(10)** 11 12 13 14 **(15)** 17		-48.95	0.89	
Item 19	1 2 3 4 5 6 7 8 9 10 11 12 13 14 15 17		-42.03	0.87	**(1)** 2 **(3)** 4 5 6 **(7) (8)** 9 **(10) (11)** 12 13 **(14)** 15 17		-55.88	0.87	
Factor Psychological Exhaustion		9.4				32.8			21.1
Item 8	1 2 3 4 5 **(6)** 7 8 9 10 11 **(12)** 13 14 15 17		-43.98	0.48	1 **(2) (3)** 4 5 **(6) (7)** 8 **(9)** 10 11 12 13 **(14) (15)** 17		-71.76	0.51	
Item 12	1 2 3 4 5 6 7 8 9 10 11 12 13 14 **(15)** 17		-40.82	0.88	1 2 **(3)** 4 5 6 **(7)** 8 9 10 11 12 13 **(14) (15)** 17		-52.92	0.73	
Item 17	1 2 3 4 5 6 7 8 9 10 11 12 13 14 **(15)** 17		-43.16	0.85	1 2 **(3)** 4 5 6 **(7)** 8 9 10 **(11)** 12 **(13) (14) (15) (17)**		-56.84	0.87	
Item 18	1 **(2)** 3 4 5 **(6)** 7 8 9 10 11 12 13 14 15 17		-41.57	0.86	1 2 3 4 5 6 **(7) (8)** 9 10 11 12 13 14 15 **(17)**		-42.95	0.96	
Factor Indolence		11.5				31.5			21.5
Item 2	1 2 3 4 5 6 7 8 **(9) (10)** 11 12 13 14 15 17		-48.82	0.54	**(1) (2)** 3 4 5 6 **(7) (8)** 9 10 **(11)** 12 13 14 **(15)** 17		-72.75	0.64	
Item 3	1 2 3 4 5 6 7 8 **(9)** 10 11 12 13 14 15 17		-49.05	0.63	1 **(2)** 3 4 5 6 7 8 9 10 11 **(12)** 13 14 **(15)** 17		-51.63	0.91	
Item 6	1 2 **(3)** 4 **(5)** 6 7 **(8)** 9 10 11 12 13 14 15 17		-59.61	0.00	1 **(2)** 3 4 **(5) (6) (7) (8) (9) (10)** 11 12 13 14 15 17		-91.72	0.39	
Item 7	1 2 **(3)** 4 5 6 7 **(8)** 9 10 **(11)** 12 13 14 **(15)** 17		-50.65	0.58	**(1) (2)** 3 4 5 6 7 8 **(9)** 10 11 **(12) (13)** 14 15 **(17)**		-57.91	0.64	
Item 11	1 2 3 4 5 6 7 8 9 10 11 12 13 14 **(15)** 17		-48.96	0.70	**(1)** 2 3 **(4)** 5 6 7 **(8)** 9 10 **(11) (12)** 13 14 15 17		-73.88	0.64	
Item 14	1 2 3 4 5 6 7 8 9 10 11 12 13 14 15 17		-42.23	0.87	1 2 3 4 **(5)** 6 7 8 9 10 11 **(12) (13)** 14 **(15)** 17		-66.53	0.62	
Factor Guilt		6.3				28.8			17.5
Item 4	1 2 3 4 5 6 7 8 9 10 11 12 **(13)** 14 15 17		-46.86	0.38	1 **(2) (3)** 4 **(5)** 6 **(7)** 8 **(9)** 10 11 **(12)** 13 14 15 **(17)**		-61.79	0.56	
Item 9	1 2 3 4 5 6 7 8 9 10 11 12 13 **(14)** 15 17		-43.42	0.71	1 2 3 4 5 6 7 8 9 10 11 12 13 **(14)** 15 **(17)**		-47.72	0.85	
Item 13	1 2 3 4 5 6 7 8 9 10 11 12 13 14 **(15)** 17		-44.35	0.77	1 2 3 4 5 **(6)** 7 8 **(9)** 10 11 **(12)** 13 14 **(15)** 17		-47.10	0.80	
Item 16	1 2 3 4 5 **(6)** 7 8 9 10 11 12 13 14 **(15)** 17		-48.19	0.00	1 2 3 **(4) (5) (6)** 7 **(8)** 9 10 **(11)** 12 **(13) (14) (15) (17)**		-62.20	0.00	
Item 20	1 2 3 4 5 6 7 8 9 10 11 12 13 14 15 17		-40.53	0.74	1 2 3 4 5 6 7 8 9 10 11 12 13 14 **(15)** 17		-42.57	0.83	

*Note*. Non-invariant parameters are bolded and parenthesized. Although one sample was excluded in the alignment analysis, the region and country labels have been kept for clarity

Country and regions labels: 1 = Argentina; 2 = Brazil; 3 = Chile; 4 = Colombia; 5 = Costa Rica; 6 = France; 7 = Italy; 8 = Spain; 9 = Poland; 10 = Puerto Rico; 11 = Valencia (Spain); 12 = Mexico; 13 = Peru; 14 = Portugal; 15 = Uruguay; 17 = Ecuador

% = percent non-invariance; All% = average percent non-invariance

**Table 5:** Comparison of the Latent Means between Countries or Regions for the Four Factors of the Spanish Burnout Inventory

Country	Mean Value	Countries with significantly smaller factor mean	Country	Mean Value	Countries with significantly smaller factor mean
Factor Enthusiasm toward the Job			Factor Psychological Exhaustion		
4 Colombia	0.098	13 11 8 5 7 12 1 2 15 14 3 9 10 6	6 France	1.313	2 3 7 12 14 9 15 8 1 11 5 13 4 17
17 Ecuador	0.000	11 8 5 7 12 2 15 14 3 9 10 6	10 Puerto Rico	1.298	2 3 7 12 14 9 15 8 1 11 5 13 4 17
13 Peru	-0.117	7 12 1 2 15 14 3 9 10 6	2 Brazil	0.823	3 7 12 14 9 15 8 1 11 5 13 4 17
11 Valencia (Spain)	-0.190	12 1 2 15 14 3 9 10 6	3 Chile	0.698	15 8 1 11 5 13 4 17
8 Spain	-0.201	12 1 2 15 14 3 9 10 6	7 Italy	0.678	15 8 1 11 5 13 4 17
5 Costa Rica	-0.229	12 2 15 14 3 9 10 6	12 Mexico	0.600	15 8 11 5 13 4 17
7 Italy	-0.281	12 2 15 14 3 9 10 6	14 Portugal	0.580	8 11 5 13 4 17
12 Mexico	-0.441	2 15 14 3 9 10 6	9 Poland	0.565	11 5 13 4 17
1 Argentina	-0.442	2 15 14 3 9 10 6	15 Uruguay	0.485	5 13 4 17
2 Brazil	-0.653	3 9 10 6	8 Spain	0.473	5 13 4 17
15 Uruguay	-0.722	3 9 10 6	1 Argentina	0.470	13 4 17
14 Portugal	-0.725	3 9 10 6	11 Valencia (Spain)	0.447	13 4 17
3 Chile	-1.190	6	5 Costa Rica	0.343	4 17
9 Poland	-1.306	6	13 Peru	0.266	17
10 Puerto Rico	-1.308	6	4 Colombia	0.208	17
6 France	-1.669		17 Ecuador	0.000	
Factor Indolence			Factor Guilt		
6 France	2.382	14 9 3 7 12 10 2 11 15 5 8 4 1 13 17	6 France	0.770	14 9 13 12 1 10 5 11 8 4 17 15 3
14 Portugal	1.519	10 2 11 15 5 8 4 1 13 17	7 Italy	0.708	2 14 9 13 12 1 10 5 11 8 4 17 15 3
9 Poland	1.422	10 2 11 15 5 8 4 1 13 17	2 Brazil	0.562	9 13 12 1 10 5 11 8 4 17 15 3
3 Chile	1.377	10 2 11 15 5 8 4 1 13 17	14 Portugal	0.484	13 12 1 10 5 11 8 4 17 15 3
7 Italy	1.326	10 2 11 15 5 8 4 1 13 17	9 Poland	0.402	5 11 8 4 17 15 3
12 Mexico	1.280	2 11 15 5 8 4 1 13 17	13 Peru	0.304	11 8 4 17 15 3
10 Puerto Rico	1.121	8 4 1 13 17	12 Mexico	0.282	11 8 4 17 15 3
2 Brazil	1.079	8 4 1 13 17	1 Argentina	0.255	4 17 15 3
11 Valencia (Spain)	1.030	8 4 1 13 17	10 Puerto Rico	0.243	8 4 17 15 3
15 Uruguay	1.005	4 1 13 17	5 Costa Rica	0.207	4 17 15 3
5 Costa Rica	1.003	4 1 13 17	11 Valencia (Spain)	0.151	4 17 15 3
8 Spain	0.906	4 1 13 17	8 Spain	0.117	17 15 3
4 Colombia	0.762	1 13 17	4 Colombia	0.039	3
1 Argentina	0.412	17	17 Ecuador	0.000	
13 Peru	0.261	17	15 Uruguay	-0.007	
17 Ecuador	0.000		3 Chile	-0.081	

As Table 4 reveals, regarding factor loadings, items 14, 15, 19, and 20 were invariant across the 16 countries and regions, whereas item 7 showed less invariance and was non-invariant in the regions of Chile (3), Spain (8), Valencia (11), and Uruguay (15). Regarding the percentage of non-invariance, the four factors obtained an average between the factor loadings and intercepts below 25%, the maximum criterion for non-invariance. The Indolence factor was shown to have the highest non-invariance across countries or regions (21.4%), whereas the Guilt factor showed the lowest percentage of non-invariance (17.5%). Regarding the fit functions, item 20 contributes the least to the fit function (-40.53). This result implies that this item is the most invariant across all the samples (it exhibits the least amount of non-invariance). In addition, the *R*^*2*^ values showed values close to 1 on the most invariant items, but some inconsistencies were found, for example, on item 15 (*R*^*2*^ = 0.77) or item 20 (*R*^*2*^ = 0.74), where *R*^*2*^ shows a lower value than other less non-invariant items (items 5, 10, 12, 17, or 18). One possible explanation for this discrepancy would be that the alignment method estimates the simplest model with the largest amount of non-invariance, but if the approximate measurement invariance assumption does not hold, the simplest and most invariant model may not be the true model (Asparouhov & Muthén, 2014). Finally, Table 5 shows the comparison of the latent means across countries or regions for the four factors of the SBI.

As the table reveals, the French sample (6) has significantly higher estimated latent means in the Psychological Exhaustion, Indolence, and Guilt factors, and the lowest in the Enthusiasm toward the job factor, compared to the rest of the countries and regions. Specifically, in the Guilt factor, the estimated latent mean shows values significantly higher than those of the other countries and regions, except Italy (7) and Brazil (2), where the difference is not statistically significant.

Another interesting result is the one observed for the Andean countries. In addition to the previously mentioned result, in the Guilt factor, the countries with lower estimated latent means are Ecuador (17), Uruguay (15), and Chile (3), with no significant differences between them. Regarding the Enthusiasm toward the job factor, Colombia (4), Peru (13), and Ecuador (17) show the highest estimated latent means. Furthermore, Colombia shows a significantly higher estimated latent mean than those of the other countries or regions, except Ecuador, with which there are no statistically significant differences. In addition, these three countries (Colombia, Peru, and Ecuador) show the lowest estimated latent means in the Psychological Exhaustion and Indolence factors, as does Argentina in the Indolence factor, with Ecuador always being the country with a significantly lower estimated latent mean in these factors.

## Discussion

The purpose of this study was to test the measurement invariance of the SBI (Gil-Monte, 2019) among professionals across 17 countries and regions. The SBI has shown a four-factor structure that remains constant in the different studies that have analyzed its psychometric properties in Latin America (e.g., Carlotto et al., 2015; García et al., 2022; Gil-Monte et al., 2013; Olivares-Faúndez et al., 2018) and in Europe (e.g., Bosle & Gil-Monte 2010; Figueiredo-Ferraz et al., 2013; Gil-Monte & Manzano-García, 2015; Misiolek et al., 2017; Viotti et al., 2015). These results indicate that the SBI offers good evidence of construct validity. However, the cultural context has an important effect on the way people from different countries and regions interpret a psychological construct. Therefore, in order to compare the measurements obtained with the SBI across countries, it is necessary to check the existence of measurement invariance. For this reason, the objective of this study was to study the cross-cultural invariance of the SBI. However, when samples from many countries are used, it is very difficult to obtain evidence of scalar measure invariance. For this reason, a different psychometric approach was used (Asparouhov & Muthén, 2014) that optimizes the estimation of means despite the presence of a certain degree of measurement non-invariance (Byrne & van de Vijver, 2017) and makes it possible to identify the most non-invariant parameters.

The results of the analyses show that the four-factor structure fits the entire sample. Likewise, the CFAs carried out with the sample from each country or region offer a good fit of the four-factor model to all the samples, except Argentina and India. The poor fit of the Indian sample to the model is probably due to the limited sample size. However, although in principle it might seem that the four-factor model does not offer a good fit in the Argentine sample due to the inconsistency between some fit indices, the results obtained in some simulation studies (Lai & Green, 2016; Shi et al., 2019, 2020) allow us to suggest that there is a good fit of the data. Therefore, only the Indian sample was excluded from the study, with 16 samples remaining: 15 countries and 1 region (Valencia, Spain).

Regarding the measurement invariance, it can be concluded that the SBI shows scalar measurement invariance, which makes it possible to compare the four factors of burnout across the sixteen samples finally included in the study. When comparing latent means by country or region, some interesting results stand out. On the one hand, the highest levels of perceived burnout are observed in the French sample because the data show the lowest scores on the Enthusiasm toward the Job factor and the highest scores on the Psychological Exhaustion, Indolence, and Guilt factors, compared to the other samples. On the other hand, some Andean countries (Colombia, Ecuador, and Peru), show the lowest levels of perceived burnout because they show the highest scores on Enthusiasm toward the Job and the lowest scores on Psychological Exhaustion and Indolence, compared to the other samples, with the exception of Colombia vs. Argentina for Indolence. In addition, in the Indolence dimension, a trend is observed, with the European countries (France, Portugal, Poland, and Italy), except Spain, showing higher levels than the Latin American countries, except Chile. Data from France, Ecuador, and Peru were collected in 2022, and so the time of data collection and the influence of job conditions as a result of the COVID-19 pandemic do not seem to explain these differences. In addition, all the participants in the samples from France, Italy, Argentina, Peru, and Ecuador were teachers. These differences could be explained by taking into consideration the different social and cultural values in Europe and Latin America. Affective values are transmitted more in Latin America, whereas moral, social, and intellectual values predominate in European education (Manzano-García & Tomé-Fernández, 2017). Moreover, studies based on Hofstede’s Cultural Dimensions Theory have concluded that individualist traits characterize people from Western Europe, whereas people from South America are described as having collectivist characteristics (Green et al., 2005), for example, France or Italy vs. Ecuador or Peru (https://www.hofstede-insights.com/country-comparison/).

Some differences observed with the samples from Chile, Colombia, Costa Rica and Poland may also be influenced by the professional characteristics of these samples, since the data from those countries were not collected in teachers (see Table 1 and Participants and Procedures section). An interesting result was the significant difference in Indolence between the Spanish sample that answered the questionnaire in Spanish and the one that answered in Catalan, given that the two samples consist of teachers working in the same geographical area (Valencian Community). The difference could be explained by the fact that the highest percentage (74.3%) of teachers who answered in Catalan were teaching in secondary school (12 to 18 years old), and the lowest percentage (25.7%) were teaching in kindergarten (3 to 6 years old) and primary school (6 to 12 years old), whereas the majority of those who responded in Spanish were teaching in kindergarten and primary school (58.8%), and the lowest percentage were teaching in secondary school (41.2%). High school students may be more demanding and show more negative and disruptive behaviors, and so teachers in secondary schools might have more discipline problems (Shaheen & Mahmood, 2016). In addition, societal respect for teachers in secondary schools seems to have declined, contributing to the development of higher levels of indolence.

### Limitations and Future Directions

In interpreting our findings, several limitations should be noted. First, sample sizes vary across countries, ranging from 210 (India), 302 (Argentina), and 316 (France) to 4,734 (Uruguay). When studying the factorial invariance of a measure between groups, these groups usually have different sizes, and when the sizes of the samples are quite unbalanced, invariance violations might not be detected because the fit function in the factorial analysis of multiple groups includes a weighting based on the sample size of the group (Yoon & Lai, 2018). However, the alignment approach we used to test for approximate measurement invariance provides an automated procedure that can overcome important limitations of traditional CFA procedures when comparing many groups. We still do not know to what extent very different sample sizes between groups can affect this method of measuring invariance.

Second, we were not able to obtain samples that are representative of their respective countries but very similar in features that might influence burnout syndrome (e.g., occupational sector, different cultures in each country). As previously mentioned, some results could be explained by the differences in the occupational sector of the participants from the different countries. Third, the data collection procedures and recruitment strategies differed by country (pencil and paper vs. online), which can produce differences across countries (see Spector et al., 2015).

As future research directions, it might be beneficial to examine measurement invariance across other demographic and occupational groups (e.g., women vs. men, health vs. education participants) in future research.

## Conclusion and Practical Implications

Our findings highlight the recommendation of using invariance testing in psychological research to better assess whether psychometric instruments are appropriate for group comparisons. Likewise, the usefulness of the alignment method to study the invariance among multiple groups has been demonstrated. The results support the validity of the SBI in the countries and regions of Europe and Latin America included in this study. We have advanced international research on burnout by introducing a reliable measure to assess it in six languages (Catalan, French, Italian, Polish, Portuguese, and Spanish) and in 16 countries and regions (excluding India) and demonstrating its psychometric qualities. We found evidence (i.e., factor validity, homogeneity within countries, and measurement invariance) that the Spanish Burnout Inventory (Gil-Monte, 2019) is a reliable and valid measure that can be used for international research projects and surveys that are concerned with research on burnout, job stress consequences, and quality of working life. This evidence is a prerequisite for identifying links between burnout levels and specific country characteristics that might help to better understand the relationship between a country’s culture and burnout.

Currently, burnout in teachers (Fernández-Suárez et al., 2021; Pressley et al., 2021; Weißenfels et al., 2022) and health professionals (Kane, 2022; Stokowski et al., 2020) is a serious problem that has increased during the COVID-19 pandemic because of the deterioration of their working conditions. Therefore, it is necessary to carry out studies to identify the evolution of this health problem and to compare the prevalence between countries and regions. To conduct these studies, psychometric instruments with sufficient measurement invariance must be applied, avoiding comparative biases due to the measurement instrument. The results of this study support the validity of the SBI to carry out these studies in the countries and regions in Europe and Latin America included in the present study.

## Data Availability

The datasets generated during and/or analysed during the current study are not publicly available due to their proprietary nature but are available from the corresponding author on reasonable request.
